# Trametinib potentiates TRAIL‐induced apoptosis via FBW7‐dependent Mcl‐1 degradation in colorectal cancer cells

**DOI:** 10.1111/jcmm.15336

**Published:** 2020-04-30

**Authors:** Lin Lin, Dapeng Ding, Xiaoguang Xiao, Bing Li, Penglong Cao, Shijun Li

**Affiliations:** ^1^ Department of Clinical Laboratory First Affiliated Hospital of Dalian Medical University Dalian China

**Keywords:** apoptosis, degradation, Mcl‐1, TRAIL, Trametinib

## Abstract

Trametinib is a MEK1/2 inhibitor and exerts anticancer activity against a variety of cancers. However, the effect of Trametinib on colorectal cancer (CRC) is not well understood. In the current study, our results demonstrate the ability of sub‐toxic doses of Trametinib to enhance TRAIL‐mediated apoptosis in CRC cells. Our findings also indicate that Trametinib and TRAIL activate caspase‐dependent apoptosis in CRC cells. Moreover, Mcl‐1 overexpression can reduce apoptosis in CRC cells treated with Trametinib with or without TRAIL. We further demonstrate that Trametinib degrades Mcl‐1 through the proteasome pathway. In addition, GSK‐3β phosphorylates Mcl‐1 at S159 and promotes Mcl‐1 degradation. The E3 ligase FBW7, known to polyubiquitinate Mcl‐1, is involved in Trametinib‐induced Mcl‐1 degradation. Taken together, these results provide the first evidence that Trametinib enhances TRAIL‐mediated apoptosis through FBW7‐dependent Mcl‐1 ubiquitination and degradation.

## 1 INTRODUCTION

Colorectal cancer (CRC) is the second most commonly reported cancer and a major cause of cancer‐related death worldwide.^1^, ^2^ The median survival time of patients with metastatic colorectal cancer has been reported to be approximately 8 months with palliative treatment, and the median survival time extends to 25.8‐31.4 months when standard chemotherapy is administered.^3^ As the major driving events of CRC progression, RAF and RAS mutations, along with TNM staging, may help in the clinical management of CRC.^4^ In addition, pathological staging and MSI status can help clinicians choose adjuvant therapy.^5^ Furthermore, the mutation states of PIK3CA, BRAF (V600E) and KRAS suggest the possibility of anti‐EGFR treatment in CRC.^6^ Current CRC treatment involves adjuvant therapy with irinotecan/oxaliplatin and 5‐fluorouracil, which increases patient survival by 1 year.^7^, ^8^ Monoclonal antibody therapy, including treatment with cetuximab and bevacizumab, has advanced CRC treatment,^9^, ^10^, ^11^ but the availability of other potent CRC drugs is lacking. New and effective anti‐CRC therapies are therefore urgently required.

Tumour necrosis factor (TNF)‐related apoptosis‐inducing ligand (TRAIL) has emerged as a promising anticancer agent.^12^, ^13^ TRAIL (part of the TNF‐α superfamily) binds to specific death receptors termed TRAIL‐R1 (DR4) and TRAIL‐R2 (DR5) to induce tumour lethality through the extrinsic and intrinsic apoptotic pathways.^12^, ^14^, ^15^ Through extrinsic signalling, TRAIL forms a multiprotein cell death signalling axis involving DR4 and DR5, FADD, and effector caspase 8.^16^ Signalling through this complex leads to the cleavage and activation of caspase‐3 and the subsequent apoptotic cell death.^16^, ^17^, ^18^ The intrinsic pathway involves cell death mediated through mitochondrial events.^18^, ^19^ TRAIL‐mediated tumour cell death can occur in response to a range of anticancer drugs.^20^ However, various cancers exhibit resistance to TRAIL, which raises questions about its efficacy as a monotherapy.^12^, ^21^ This resistance can be circumvented through dual therapies that sensitize cancer cells to TRAIL, but the discovery of such agents is challenging.^22^ It has been reported that Bcl‐2 and Bcl‐xL inhibitors can enhance cancer cell sensitivity to TRAIL.^23^, ^24^

Trametinib (Mekinist) is a selective MEK1/2 inhibitor with activity against BRAF V600 melanomas.^25^, ^26^ Trametinib is currently approved for cases of metastatic, unresectable melanomas harbouring the BRAF‐V600E/K mutation and can be combined with dabrafenib to improve its therapeutic efficacy.^27^, ^28^ However, the effect and mechanism of Trametinib on CRC cells have not been well studied.

Mcl‐1 is a very unstable protein, and the degradation of Mcl‐1 can be triggered by a variety of stresses, including anticancer drugs.^29^ Mcl‐1 protein stability and activity are regulated by posttranslational modifications, such as phosphorylation.^30^ The Mcl‐1 protein contains a proline/glutamic acid/serine/threonine (PEST) region, which is phosphorylated.^31^ Glycogen synthase kinase 3β (GSK‐3β) or other kinases that phosphorylate Mcl‐1 promote Mcl‐1 binding to E3 ubiquitin ligases, including Mule, FBW7 (F‐box and WD repeat domain‐containing 7), and β‐TrCP, leading to Mcl‐1 ubiquitination and its subsequent proteasomal degradation.^32^, ^33^ Multiple studies using overexpressed Mcl‐1 mutants have shown that Mcl‐1 phosphorylation also affects its anti‐apoptotic activity and interactions with other Bcl‐2 family proteins.^7^, ^8^

Here, we assessed the ability of Trametinib to sensitize CRC tumours to TRAIL‐mediated cell death in CRC. We provide the first evidence of the ability of Trametinib to enhance CRC apoptosis in combination with TRAIL, and this effect is mediated by Mcl‐1 degradation.

## 2 MATERIALS AND METHODS

### 2.1 Cell culture

Colorectal cancer cell lines including DLD1, RKO, HT29 and HCT116 were obtained from the American Type Culture Collection (ATCC, Manassas, VA, USA). Normal colonic epithelial NCM356 cells were obtained from INCELL (San Antonio, TX, USA). The cells were grown in RPMI 1640 plus FBS (10%), 100 U/mL penicillin and 100 μg/mL streptomycin (Invitrogen, Carlsbad, CA, USA) at 37°C in 5% CO_2_. Trametinib was obtained from Selleckchem (Houston, TX, USA), and human TRAIL (recombinant) was obtained from Sigma (St. Louis, MO, USA). For drug treatment, the cells were plated in 12‐well plates at a density of 20%‐30% 24 hours before treatment.

### 2.2 Gene silencing (siRNA)

siRNAs against Mcl‐1 (sc‐35877) and FBW7 (sc‐37547) and a control siRNA (scrambled; sc‐37007) were obtained from Santa Cruz Biotechnology (Dallas, TX, USA). The indicated cells were seeded in 12‐well plates for 24 hours. The Lipofectamine RNAi Max reagent (Invitrogen) was used for siRNA transfections for 24 hours. The cells were treated with Trametinib/TRAIL for 24 hours for further analysis.

### 2.3 Transfection

For the overexpression studies, the human Flag‐tagged Mcl‐1 pcDNA3.1 or empty vector controls were obtained from Addgene (Cambridge, MA, USA). The mutations were introduced into Mcl‐1 using the QuickChange XL Site‐Directed Mutagenesis Kit (Agilent Technologies, Santa Clara, CA, USA). Transfection was performed using Lipofectamine 2000 (Invitrogen) according to the manufacturer's instructions.

### 2.4 MTT assay

To assess the viability, 1 × 10^4^ cells in 96‐well flat‐bottom plates were treated with increasing concentrations of Trametinib or TRAIL as indicated for 72 hours. To each well, 20 μL of 5 mg/mL MTT reagent (Roche, Basel, Switzerland) was added, and the plates were incubated for 1 hour in the tissue culture incubator at 37°C. The crystals of formazan were solubilized with 150 μL DMSO after the media were removed. The absorbance at 450 nm was determined by microplate reader.

### 2.5 Colony formation assay

For the colony‐forming assays, the HCT116 cells were treated with Trametinib, TRAIL or their combination for 24 hours, plated in 12‐well plates at equal numbers (500 cells) and cultured for 2 weeks. The cells were washed with PBS, and the colonies were fixed (methanol ~95%) and stained using crystal violet solution.

### 2.6 Assessment of apoptosis

Apoptotic cells were identified using the FITC Annexin V/PI Apoptosis Detection Kit, and fragmented nuclei were assessed through Hoechst 33258 staining. Briefly, the cells were exposed to Trametinib/TRAIL for 24 hours in binding buffer and labelled Annexin V‐FITC was added for 15 minutes. The apoptotic cells were assessed by flow cytometry (BD FACSCanto II).

### 2.7 Western blotting

Western blotting was performed as previously described.^2^, ^34^ Briefly, the indicated cells treated with Trametinib and/or TRAIL were lysed in RIPA buffer plus protease inhibitors. The proteins were separated by 10% sodium dodecyl sulphate‐polyacrylamide gel electrophoresis and wet‐transferred onto polyvinylidene fluoride membranes (PVDF membranes; Amersham Bioscience, Piscataway, NJ, USA). The membranes were blocked in 5% BSA in PBS‐T to prevent nonspecific antibody binding. The proteins were labelled with primary antibodies (4°C, ON) and labelled with the appropriate HRP‐conjugated secondary antibodies (1 hour, RT). The bands were detected using enhanced Chemiluminescence Kits (Amersham Bioscience). The antibodies were as follows: PUMA, cleaved caspase 3, cleaved caspase 8, cleaved caspase 9, cleaved PARP, β‐actin, and p‐Mcl‐1 (S159) (Cell signalling technology); Bcl‐2, Bcl‐XL, Bim, Noxa, and Survivin (Abcam); and HA, V5, FBW7, Mcl‐1 and Bax (Santa Cruz Biotechnology).

### 2.8 Real‐time quantitative PCR

RNA was extracted from the HCT116 cells treated with Trametinib at the indicated time points using TRIzol reagent following the manufacturer's instructions. The SuperScript II RT Kit was used for cDNA synthesis. Real‐time PCR was performed with SsoFasr^TM^ Probes Supermix (Bio‐Rad, Hercules, CA, USA) on a Bio‐Rad CFX96^TM^ RT‐PCR System (35 cycles). The expression levels were evaluated by TaqMan RT‐PCR, and the results were plotted as the threshold cycle (Ct). The relative abundance of the target genes was determined using the comparative Ct method (ΔΔCt). Gene expression was assessed using the 2^−ΔΔCt^ method. Primers: Mcl‐1, Forward: 5′‐GACCTGACAGACTACCTCAT‐3′, Reverse: 5′‐AGACAGCACTGTGTTGGCTA‐3′; and β‐actin, Forward: 5′‐ATGCTTCGGAAACTGGACAT‐3′, Reverse: 5′‐TGGAAGAACTCCACAAACCCA‐3′.

### 2.9 Co‐immunoprecipitation

For Co‐IP assays, the cells were lysed by scrape‐harvesting and suspended in 1 mL of lysis buffer (50 mmol/L Tris‐HCl, pH 7.5, 100 mmol/L NaCl, 0.5% Nonidet P‐40) supplemented with a protease inhibitor cocktail (Sigma). The cell lysates were collected and centrifuged for 5 minutes at 12 600 *g* (4°C). The clarified lysates were labelled with 2 μg of primary antibodies (ON, 4°C) followed by the addition of protein G beads for 1 hour at 4°C. The beads were then washed with cold lysis buffer and centrifuged. The bound proteins were extracted from the beads using 2× Lamelli buffer and assessed by Western blot assay.

### 2.10 Statistical analysis

Statistical analysis was carried out using GraphPad InStat V software (GraphPad Software Inc., San Diego, CA, USA). The results are expressed as the mean of arbitrary values ± SD. All the results were evaluated using unpaired Student's *t* test. *P* < 0.05 was considered significant.

## 3 RESULTS

### 3.1 Trametinib/TRAIL synergistically promote CRC apoptosis

**Figure 1:**
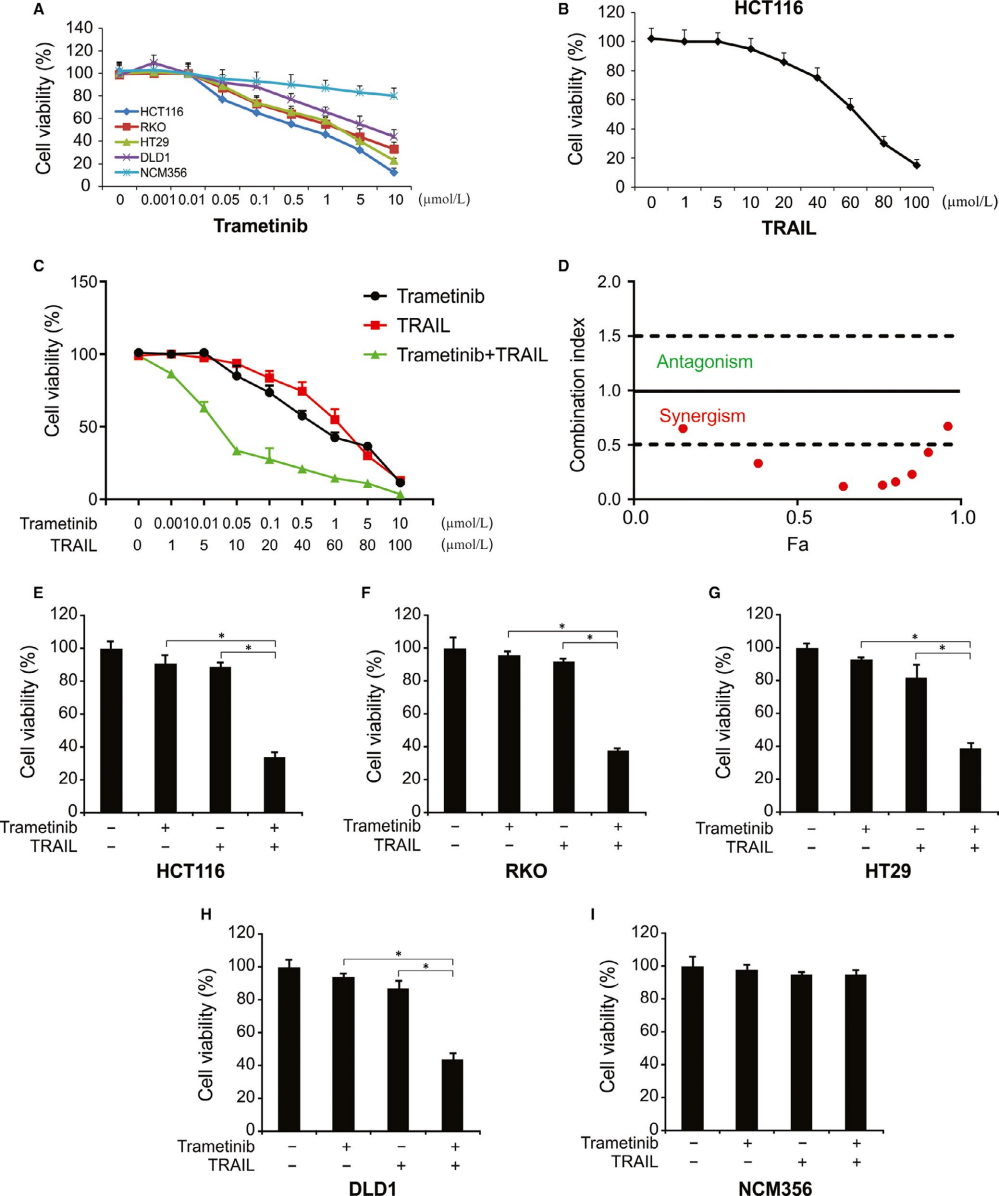
Trametinib promotes TRAIL‐induced cytotoxicity of human CRC cell lines. A, Indicated cells were treated with various concentrations of Trametinib for 72 h. Cell growth was analysed by MTT. B, HCT116 cells were treated with various concentrations of TRAIL for 72 h. Cell growth was analysed by MTT. C, HCT116 cells were treated with Trametinib and TRAIL at indicated concentration for 72 h. Cell growth was analysed by MTT. D, Combination index is shown for HCT116 cells. Fa, fraction affected. E‐I, Indicated cells were incubated in the presence or absence of TRAIL (10 ng/mL) and/or Trametinib (0.1 μmol/L) for 72 h. Cell growth was analysed by MTT. Results in (E)‐(I) were expressed as means ± SD of three independent experiments. *, *P* < 0.05

Trametinib is known to induce cell death in melanoma, leukaemia and lung cancer cells.^35^, ^36^, ^37^ We first investigated whether Trametinib can induce cell death independently of TRAIL in CRC cells. In the experiments that followed, the CRC cell lines were treated with increasing doses of Trametinib for 72 hours, and the CRC cell viability was assessed by MTT. We observed that various levels of Trametinib sensitivity existed in all the CRC cells assessed, while little loss of NCM356 cell viability was observed at concentrations as high as 10 μmol/L (Figure 1A). Since HCT116 cells are more sensitive than other cell lines, we selected this cell line for the subsequent experiments. Moreover, we analysed the effect of TRAIL on HCT116 cells. We found that the IC50 was higher than 50 ng/mL (Figure 1B). For the combination, we found strong synergistic effects of Trametinib and TRAIL in HCT116 cells (Figure 1C). The combination index (CI) values of Trametinib and TRAIL are shown in Figure 1D. Thus, for the following experiments, we used 10 ng/mL for the next combination treatments. When Trametinib and TRAIL were combined, higher levels of cytotoxicity were observed in HCT116 and DLD1, RKO and HT29 cells (Figure 1E‐H), while NCM356 cells showed minimal losses in viability (Figure 1I); those outcomes indicates that the combination treatment has little cytotoxicity on normal cells. Taken together, these data demonstrate that cotreatment with Trametinib plus TRAIL sensitizes CRC cells and induces cytotoxicity.

### 3.2 Trametinib enhances TRAIL‐mediated apoptosis

**Figure 2:**
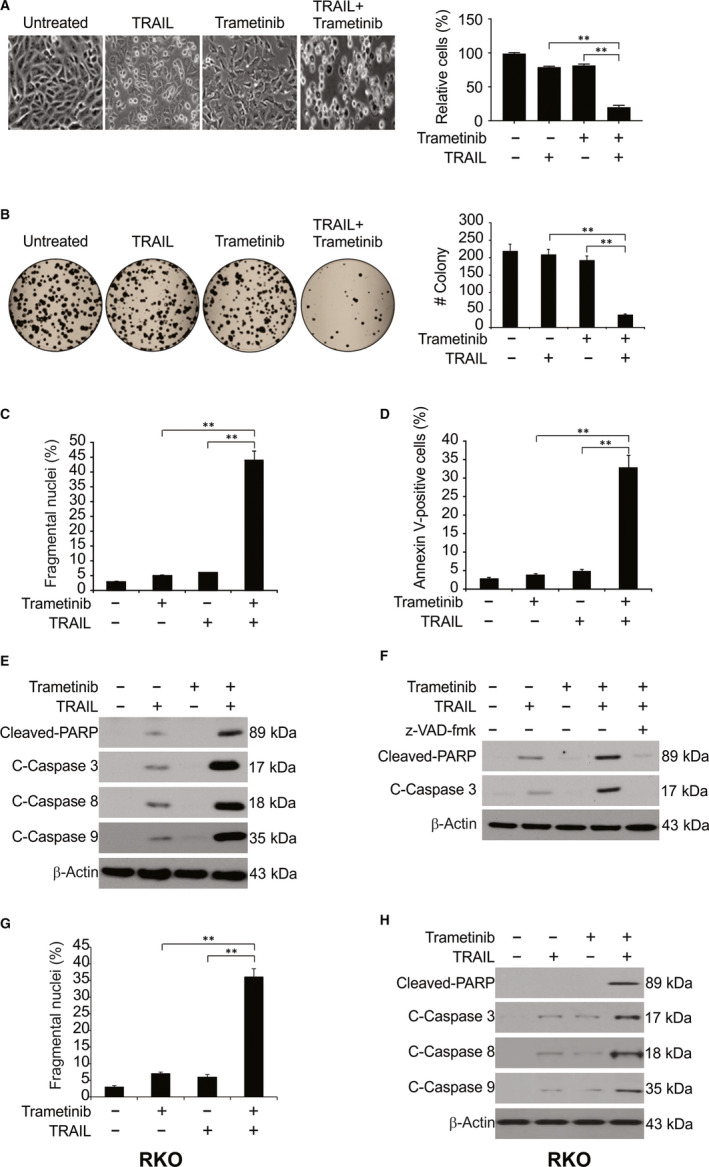
Trametinib sensitizes TRAIL‐induced apoptosis in CRC cells. A, HCT116 cells were treated with 0.1 μmol/L Trametinib, 10 ng/mL TRAIL or their combination 24 h. Cell morphology was examined under a light microscope. Attached cells were counted. B, HCT116 cells plated in six‐well cell culture plates were treated with 0.1 μmol/L Trametinib, 10 ng/mL TRAIL, or their combination for 24 h. After 14 days, the plates were stained for cell colonies with crystal violet dye, and photographs of colonies taken using a digital camera. C, HCT116 cells were treated with 0.1 μmol/L Trametinib, 10 ng/mL TRAIL or their combination for 24 h. Apoptosis was analysed by a nuclear fragmentation assay. D, HCT116 cells were treated with 0.1 μmol/L Trametinib, 10 ng/mL TRAIL, or their combination for 24 h. Apoptosis was analysed by Annexin V/PI staining followed by flow cytometry. E, HCT116 cells were treated with 0.1 μmol/L Trametinib, 10 ng/mL TRAIL, or their combination for 24 h. Indicated proteins were analysed by Western blotting. F, HCT116 cells pre‐treated with 10 μmol/L z‐VAD‐fmk for 1 h were treated with 0.1 μmol/L Trametinib, 10 ng/mL TRAIL, or their combination for 24 h. Indicated proteins were analysed by Western blotting. G, RKO cells were treated with 0.1 μmol/L Trametinib, 10 ng/mL TRAIL or their combination for 24 h. Apoptosis was analysed by a nuclear fragmentation assay. H, RKO cells were treated with 0.1 μmol/L Trametinib, 10 ng/mL TRAIL or their combination for 24 h. Indicated proteins were analysed by Western blotting. Results in (B), (C), (D) and (G) were expressed as means ± SD of three independent experiments. **, *P* < 0.01

We next assessed the synergism of Trametinib/TRAIL by investigating their effects on HCT116 morphology (Figure 2A). We observed gross morphological changes in cells cotreated with Trametinib and TRAIL (Figure 2A). We also assessed the long‐term effects on cell survival in response to Trametinib and/or TRAIL by CFAs. The combination of the two drugs potently reduced colony numbers to a higher degree that the effect of either drug alone, confirming a synergistic effect (Figure 2B). To investigate whether the loss of cell viability was due to induction of apoptosis, we assessed the expression of apoptotic markers in Trametinib/TRAIL‐treated cells. As shown in Figure 2C,D, Trametinib promoted TRAIL‐induced apoptosis in HCT116 cells. Trametinib also promoted the effects of TRAIL on caspases 3, 8, 9 and increased the cleavage of PARP (Figure 2E). The caspase inhibitor z‐VAD‐fmk attenuated the Trametinib/TRAIL‐induced PARP cleavage (Figure 2F). Moreover, enhanced apoptosis by the Trametinib/TRAIL combination was also observed in RKO cells (Figure 2G,H). Thus, Trametinib enhances TRAIL‐induced apoptosis through the induction of extrinsic and intrinsic apoptosis.

### 3.3 Trametinib down‐regulates Mcl‐1 to sensitize CRC cells to TRAIL

**Figure 3:**
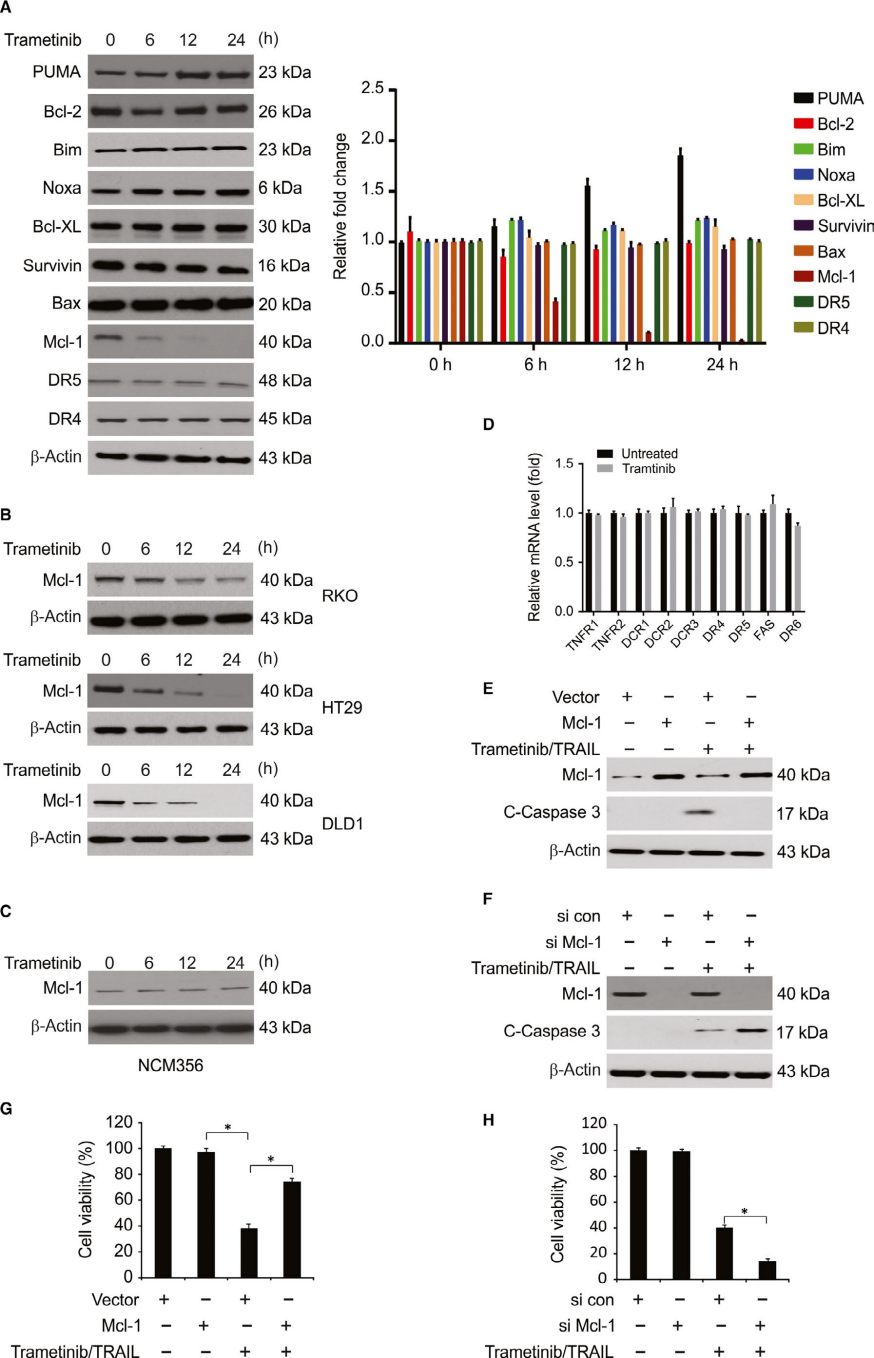
Trametinib‐induced Mcl‐1 down‐regulation is associated with the induction of TRAIL‐mediated apoptosis. A, HCT116 cells were treated with 0.1 μmol/L Trametinib at indicated time point. Indicated protein level was determined by Western blotting and normalized to β‐actin. B, Indicated cell lines were treated with 0.1 μmol/L Trametinib at indicated time point. Mcl‐1 level was analysed by Western blotting. C, NCM356 cells were treated with 0.1 μmol/L Trametinib at indicated time point. Mcl‐1 level was analysed by Western blotting. D, HCT116 cells were treated with 0.1 μmol/L Trametinib for 24 h. Relative mRNA levels of indicated gene were analysed by real‐time PCR. E, HCT116 cells transfected with Mcl‐1 were treated with the combination of 0.1 μmol/L Trametinib and 10 ng/mL TRAIL for 24 h. Cleaved caspase 3 was analysed by Western blotting. F, HCT116 cells transfected with si control or si *Mcl‐1* were treated with the combination of 0.1 μmol/L Trametinib and 10 ng/mL TRAIL for 24 h. Cleaved caspase 3 was analysed by Western blotting. G, HCT116 cells transfected with Mcl‐1 were treated with the combination of 0.1 μmol/L Trametinib and 10 ng/mL TRAIL for 72 h. Cell growth was analysed by MTT. H, HCT116 cells transfected with si control or si *Mcl‐1* were treated with the combination of 0.1 μmol/L Trametinib and 10 ng/mL TRAIL for 72 h. Cell growth was analysed by MTT. Results in (D), (G) and (H) were expressed as means ± SD of three independent experiments. *, *P* < 0.05

We next investigated whether Trametinib sensitizes HCT116 cells to TRAIL through the stimulation of death receptor pathways. As shown in Figure 3A, we observed no changes in the expression of survivin, Bcl‐XL, Bcl‐2, Bax, DR4 and DR5 in response to Trametinib treatment. However, we observed a marked decrease in Mcl‐1 expression in response to Trametinib treatment (Figure 3A) in all the tested CRC cells (Figure 3B). Our findings also show that Trametinib treatment does not down‐regulate the Mcl‐1 levels in NCM356 cells (Figure 3C). We also examined the effect of Trametinib on the mRNA levels of DR4, DR5 and other TNF receptor superfamily members. No changes in the mRNA levels of DR4, DR5 and the other TNF receptor superfamily members were observed in HCT116 cells after Trametinib treatment (Figure 3D). To confirm its importance, we investigated the effects of Mcl‐1 overexpression in HCT116 cells. As shown in Figure 3E,G, the combination of Trametinib and TRAIL‐induced apoptosis and growth inhibition were significantly attenuated by Mcl‐1 overexpression. The silencing of Mcl‐1 produced the opposite phenotype and enhanced cell death in response to Trametinib/TRAIL treatment (Figure 3F,H). Our findings demonstrated that Trametinib exerts its effects by decreasing Mcl‐1 expression.

### 3.4 Trametinib induces Mcl‐1 degradation in a ubiquitin‐proteasome manner

**Figure 4:**
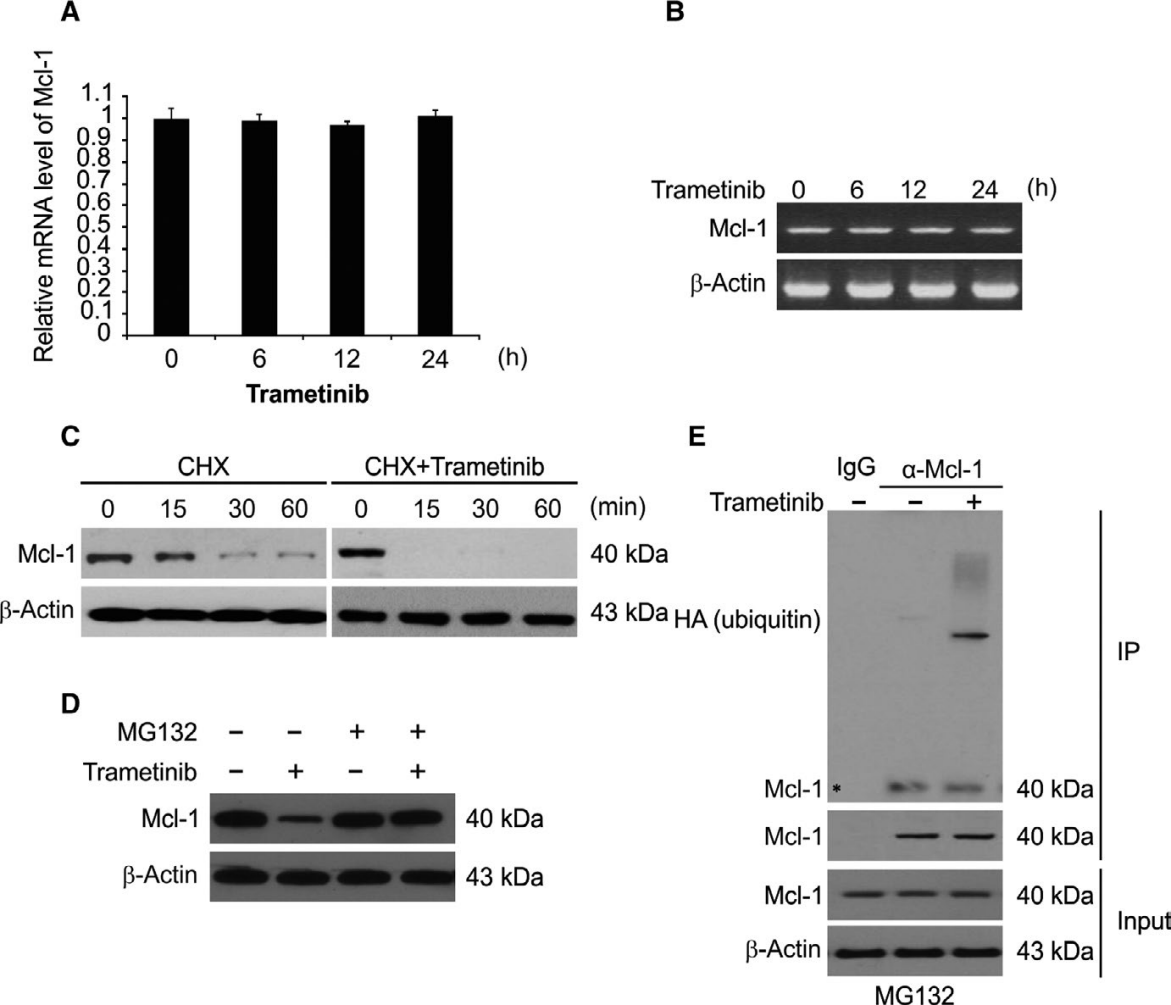
Trametinib promotes Mcl‐1 degradation and ubiquitination. A, HCT116 cells were treated with 0.1 μmol/L Trametinib at indicated time point. *Mcl‐1* mRNA level was analysed by RT‐qPCR, with β‐actin as a control. B, HCT116 cells were treated with 0.1 μmol/L Trametinib at indicated time point. Total RNA was extracted, and *Mcl‐1* mRNA expression was analysed by semiquantitative reverse transcription PCR, followed by gel electrophoresis. β‐actin was used as a control. C, HCT116 cells were treated with or without Trametinib in the presence of cyclohexamide (CHX) (10 μg/mL) for the indicated time periods. The Mcl‐1 protein level was determined by Western blotting. D, Trametinib‐treated cells were treated with or without MG132 and subjected to Western blotting. E, HCT116 cells transfected with HA‐ubiquitin and pre‐treated with 5 μmol/L MG132 for 30 min were treated 0.1 μmol/L Trametinib for 4 h. IP was performed to pull down Mcl‐1, followed by Western blotting of indicated proteins

Given these findings, we further examined the relationship between Trametinib and Mcl‐1. Thus, we evaluated whether Trametinib regulates the mRNA level of Mcl‐1. The real‐time quantitative PCR (RT‐qPCR) (Figure 4A) and semiquantitative RT‐PCR (Figure 4B) results showed no change in the Mcl‐1 mRNA levels in response to Trametinib treatment. We next examined the effects of Trametinib on protein stability. When HCT116 cells were exposed to Trametinib and cyclohexamide (CHX, 10 μg/mL), Trametinib decreased the Mcl‐1 protein stability in HCT116 cells (Figure 4C). Furthermore, after treatment with CHX, the rate of Mcl‐1 degradation was significantly greater in Trametinib‐treated cells than that in untreated cells. Moreover, previous studies have shown that Mcl‐1 degradation is generally regulated by the ubiquitin‐proteasome pathway.^30^ Therefore, we next assessed the influence of MG132, a proteasome inhibitor, on Trametinib‐induced Mcl‐1 degradation. Figure 4D shows that MG132 significantly inhibited Mcl‐1 degradation in response to Trametinib. Our findings also showed that Trametinib promoted Mcl‐1 ubiquitination in HCT116 cells (Figure 4E). The data described above indicate that Trametinib down‐regulates Mcl‐1 levels in a ubiquitin‐proteasome‐dependent manner.

### 3.5 Trametinib enhances the Mcl‐1 and FBW7 interaction in CRC cells

**Figure 5:**
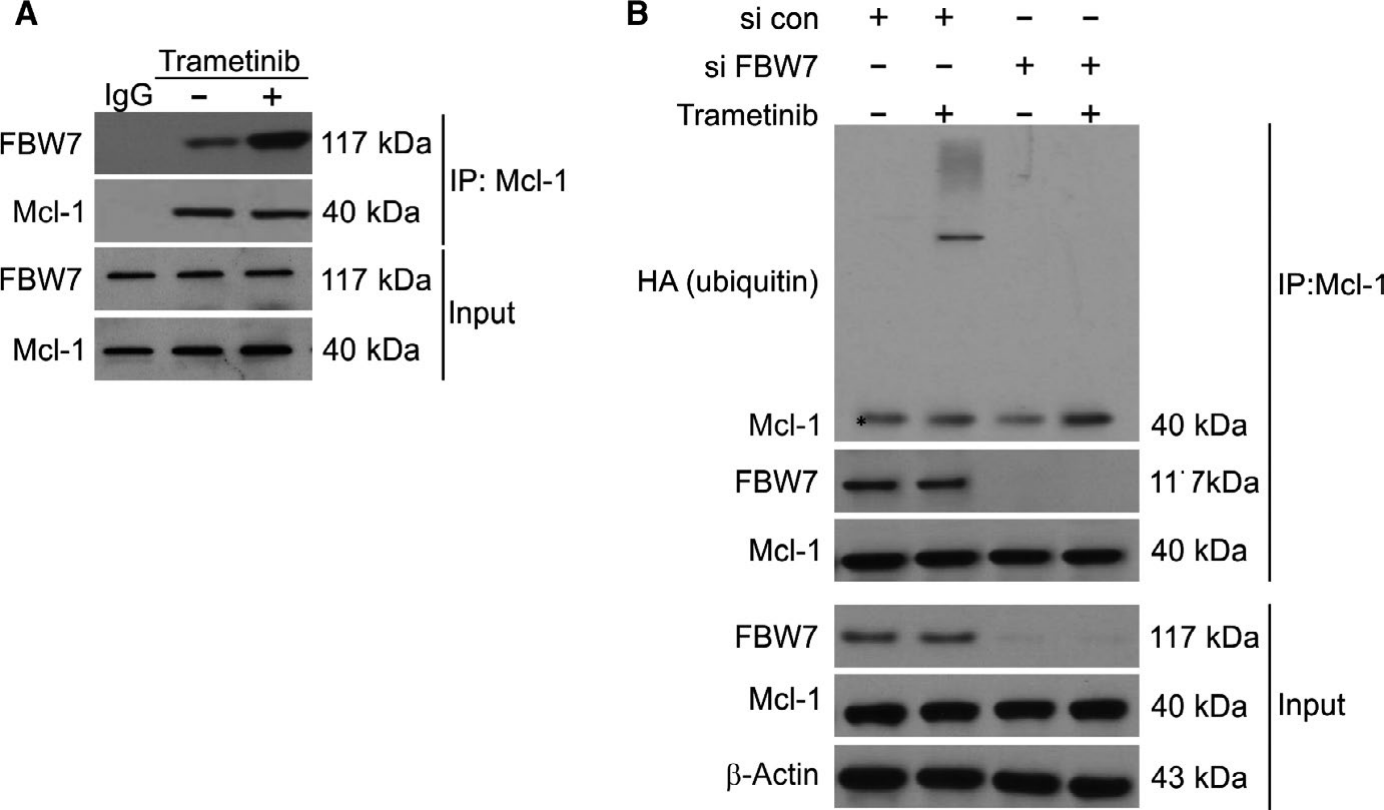
FBW7 is required for Trametinib‐induced Mcl‐1 degradation and ubiquitination. A, HCT116 cells were treated with 0.1 μmol/L Trametinib for 24 h. IP was performed to pull down Mcl‐1, followed by Western blotting of indicated proteins. B, Parental and FBW7 knockdown HCT116 cells transfected with HA‐ubiquitin and pre‐treated with 5 μmol/L MG132 for 30 min were treated 0.1 μmol/L Trametinib for 4 h. IP was performed to pull down Mcl‐1, followed by Western blotting of indicated proteins

FBW7 is an E3 ligase known to ubiquitinate Mcl‐1 and target it for proteasomal degradation.^38^ We therefore investigated the effect of Trametinib on Mcl‐1 and FBW7 binding by co‐IP assays. We observed an enhanced interaction of Mcl‐1 and FBW7 following Trametinib treatment (Figure 5A). We also found that the ubiquitination of Mcl‐1 was absent in FBW7 knockdown cells (Figure 5B). Taken together, these data demonstrated that Trametinib enhances the interaction of FBW7 with Mcl‐1 to mediate Mcl‐1 degradation.

### 3.6 GSK‐3β mediates Trametinib‐induced Mcl‐1 degradation

**Figure 6:**
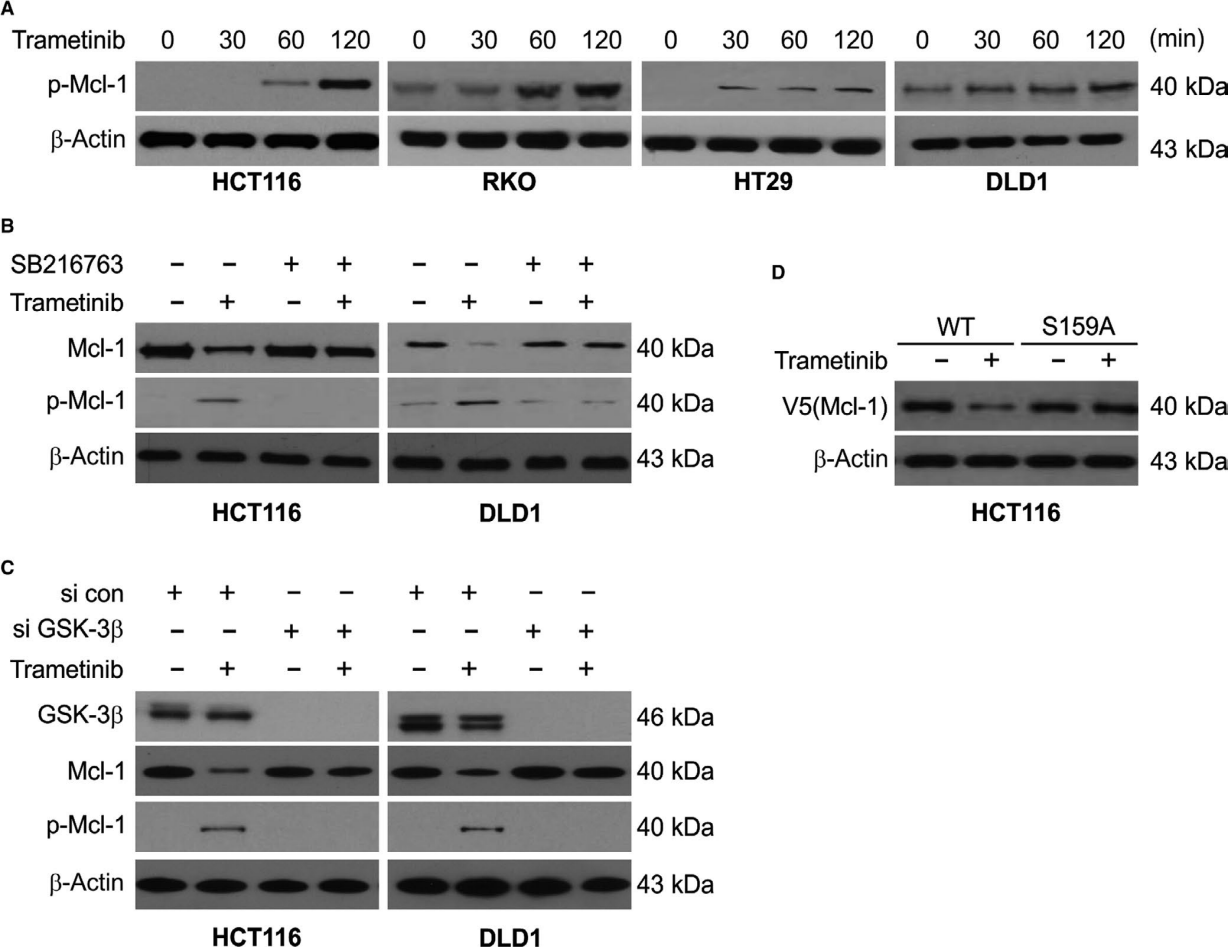
GSK3β mediates Trametinib‐induced Mcl‐1 phosphorylation and degradation. A, Indicated cell lines were treated with 0.1 μmol/L Trametinib at indicated time point. Phosphorylation of Mcl‐1 was analysed by Western blotting. B, HCT116 and DLD1 cells were pre‐treated with 1 μmol/L SB216763 for 1 h and then treated with 1 μmol/L Trametinib for an additional 2 h. Indicated protein level was determined by Western blotting. C, HCT116 and DLD1 cells transfected with si control or *GSK3β* siRNA were treated with 0.1 μmol/L Trametinib for an additional 2 h. Indicated protein level was determined by Western blotting. D, HCT116 cells transfected with WT or S159A Mcl‐1. After 24 h, the cells were treated with 0.1 μmol/L Trametinib for an additional 2 h. Indicated protein level was determined by Western blotting

Previous studies have shown that phosphorylation of Mcl‐1 by GSK‐3β at S159 leads to its down‐regulation.^30^, ^38^ We next detected the Mcl‐1 phosphorylation levels at this site in Trametinib‐treated cells. As early as 30 minutes post‐Trametinib treatment, we observed a rapid enhancement of phosphorylation at S159 (Figure 6A) suggesting a GSK3β‐dependent Mcl‐1 reduction. To confirm this observation, we assessed the effects of Trametinib in the presence of the chemical GSK3β inhibitor SB216763. We found that SB216763 inhibited the Trametinib‐stimulated Mcl‐1 phosphorylation and degradation in HCT116 and DLD1 cells (Figure 6B). In agreement with this finding, GSK3β silencing also inhibited the effects of Trametinib on Mcl‐1 (Figure 6C). We also observed a reduced ability of Trametinib to degrade Mcl‐1 when S159 of Mcl‐1 was mutated to S159A (Figure 6D). Taken together, these data revealed that pS159 of Mcl‐1 is required for its Trametinib‐stimulated degradation.

## 4 DISCUSSION

Trametinib is a highly selective MAPK kinase (MEK) 1/2 allosteric inhibitor. Trametinib inhibits ERK1/2 phosphorylation.^39^ In clinical practice, BRAF inhibitors, including trametinib and dabrafenib, are widely used to treat and prevent metastatic melanoma.^40^ However, the function of Trametinib in CRC is not well understood. Previous studies have shown that TRAIL can reduce the aggressiveness of colon cancers and promote the apoptosis of colon carcinoma cells.^41^, ^42^ In this study, the combined inhibitory effects of Trametinib and TRAIL were studied, and the potential mechanism by which Trametinib increases the sensitivity of CRC cells to TRAIL was explored. Here, we have shown that Trametinib and TRAIL synergistically stimulate apoptosis in CRC cells by degrading Mcl‐1. Thus, Trametinib exhibits a multifactorial mechanism of promoting TRAIL lethality. Mcl‐1, a well‐known Bcl‐2 family protein, negatively regulates apoptosis by binding and sequestering pro‐apoptotic proteins, including Bak, Noxa, Bax, PUMA and Bim.^43^, ^44^ Mcl‐1 also plays a role in CRC progression.^8^, ^45^ In this study, we found that Trametinib enhances GSK3β‐mediated Mcl‐1 phosphorylation at S159. We also found that GSK3β is involved in Trametinib‐induced Mcl‐1 degradation. We showed that the loss of Mcl‐1 in response to Trametinib could be inhibited by the proteasome inhibitor MG132, but no changes were observed in the Mcl‐1 mRNA levels. This is an important finding, since Mcl‐1 expression is related to tumour recurrence and reduced survival rates in CRC patients. In addition, enhanced expression of anti‐apoptotic proteins has been observed in TRAIL‐resistant cancers. We observed that Mcl‐1 overexpression could inhibit Trametinib/TRAIL‐induced tumour cell death, while decreased Mcl‐1 increased Trametinib/TRAIL‐dependent apoptosis. These findings emphasized the role of Mcl‐1 in Trametinib/TRAIL‐induced apoptosis and its potential role in the resistance of CRC cells to anticancer therapies.

FBW7 is the substrate recognition component of the evolutionarily conserved SCF‐type ubiquitin ligase.^46^ FBW7 degrades some proto‐oncogenes that play a role in cell growth and division pathways, including JUN, Notch, MYC and cyclin E.^47^ FBW7 is also a tumour suppressor and its regulatory network is disrupted in many human malignancies.^48^ Many cancer‐related mutations in FBW7 and its substrates have been identified, and loss of FBW7 function leads to chromosomal instability and tumorigenesis.^49^ In the current study, we have been suggested that Trametinib inhibits the stability of Mcl‐1 in CRC by influencing its ubiquitination. In agreement with this hypothesis, Trametinib was found to enhance the binding of Mcl‐1 to FBW7. FBW7 has been recently suggested to be a key E3 ligase that mediates GSK3‐dependent Mcl‐1 degradation.^45^ A role of FBW7 was confirmed in silencing experiments, in which a lack of FBW7 protected Mcl‐1 from Trametinib‐mediated degradation. The ability of MG132 to block Trametinib‐mediated Mcl‐1 degradation was further confirmed by the stabilization of Mcl‐1 following treatment with MG132. These findings confirmed that Trametinib regulates Mcl‐1 expression by regulating its degradation.

The phosphorylation of Mcl‐1 at S159 has been previously identified as a key signal that depends on GSK‐3β to degrade S159 phosphorylated Mcl‐1.^50^ Our findings show that FBW7‐mediated Mcl‐1 degradation requires S159 phosphorylation and GSK‐3β activation. In addition, S159 phosphorylation regulates the binding of FBW7 to Mcl‐1. Overall, these results indicate that FBW7 mediates the S159 phosphorylation‐dependent Mcl‐1 protein turnover.

In summary, our findings demonstrate a new mechanism of Trametinib in sensitization to TRAIL. These findings add new knowledge to our understanding of the role of Trametinib in the pathophysiology and treatment of CRC. To the best of our knowledge, these are the first data to reveal that Trametinib enhances TRAIL sensitization by targeting Mcl‐1 via ubiquitin‐proteasome degradation.

## CONFLICT OF INTEREST

The authors have declared that no conflicts of interest exist.

## AUTHOR'S CONTRIBUTION

Lin Lin, Dapeng Ding and Shijun Li contributed to the conception of the manuscript. Lin Lin, Dapeng Ding, Xiaoguang Xiao, Bing Li and Penglong Cao performed the experiments. Lin Lin, Dapeng Ding and Shijun Li drafted and revised the manuscript. All authors read and approved the final manuscript.

## Data Availability

The data that support the findings of this study are available from the corresponding author upon reasonable request.
